# Death of Prof. R. L. Howard

**Published:** 1854-03

**Authors:** 


*Death of Prof. R. L. Howard.*—This distinguished gentleman, the late professor of Surgery in the Starling Medical College, has, since our last issue, been taken from the field of his labors by death.

His disease was albuminuria, and on the autopsy it was found that he had also fatty liver. The immediate cause of death, however, was double pneumonia, not of an active grade, but still too much for his enfeebled powers to recover from.

Dr. Howard's life was characterized by industry in his calling, and intense devotion to medicine as a science. No man labored harder for a good name among physicians; no man won his laurels in a fairer or more honorable way. As editor of the Ohio Medical Journal, his writings were marked by force and vigor of style, and gave evidence of study in their preparation. We trust that some one familiar with his life, may prepare such a memoir as may perpetuate his memory and his good deeds.

Two men have died from the Starling faculty just at the moment when life was most desirable. All who knew Dr. John Butterfield, the first Professor of Theory and Practice in that school, must recollect the beauty of his life, the elegance of his writings, and his noble qualities as a teacher. Like Howard, his position was but fairly established—the fame for which he labored but just within his grasp—when that mysterious Providence, which regards not the plans of men, removed him from his usefulness.

The chair of surgery in the Starling school is now vacant. The hiatus caused by the illness and death of Dr. Howard, has been filled during the past winter by Prof. E. M. Moore, of the chair of anatomy in the Buffalo school, and, as we learn, most efficiently and satisfactorily to the class. But no voucher is required, for Dr. Moore's ability as a teacher, by those who know or have once heard him. H.

